# Characteristics of Tibetan pig lung tissue in response to a hypoxic environment on the Qinghai–Tibet Plateau

**DOI:** 10.5194/aab-64-283-2021

**Published:** 2021-06-28

**Authors:** Yanan Yang, Caixia Gao, Tianliang Yang, Yuzhu Sha, Yuan Cai, Xinrong Wang, Qiaoli Yang, Chengze Liu, Biao Wang, Shengguo Zhao

**Affiliations:** 1 College of Animal Science and Technology, Gansu Agricultural University, Lanzhou 730070, China; 2 State Key Laboratory of Veterinary Biotechnology, Harbin Veterinary Research Institute, Chinese Academy of Agricultural Sciences, Harbin 150069, China

## Abstract

To adapt to the plateau environment, Tibetan pigs' lungs have developed a unique physiological mechanism during evolution. The vascular corrosion casting technique and scanning electron microscopy were used to understand arterial architecture. Blood physiological index and quantitative real-time PCR (qRT-PCR) were used for assessing whether the lung can regulate the body through anatomical, physiological and molecular mechanisms to adapt to hypoxic environments. Our study showed that the lungs of Tibetan pigs were heavier and wider and that the pulmonary arteries were thicker and branched and had a denser vascular network than those of Landrace pigs. The hemoglobin (HGB), mean corpuscular hemoglobin concentration (MCHC) values of high-altitude pigs were significantly higher than those of low-altitude pigs. The expression levels of *HIF*- *1* α , *EPAS1*, *EPO* and *VEGF*, but not those of *eNOS*and *EGLN1*, were significantly higher in the lungs of high-altitude pigs than in those from pigs at a lower altitude ( P<0.05 ). These findings and a comprehensive analysis help elucidate the pulmonary mechanism of hypoxic adaptation in pigs.

## Supplement

10.5194/aab-64-283-2021-supplement The supplement related to this article is available online at: https://doi.org/10.5194/aab-64-283-2021-supplement.

## Figures and Tables

**Figure 1:**
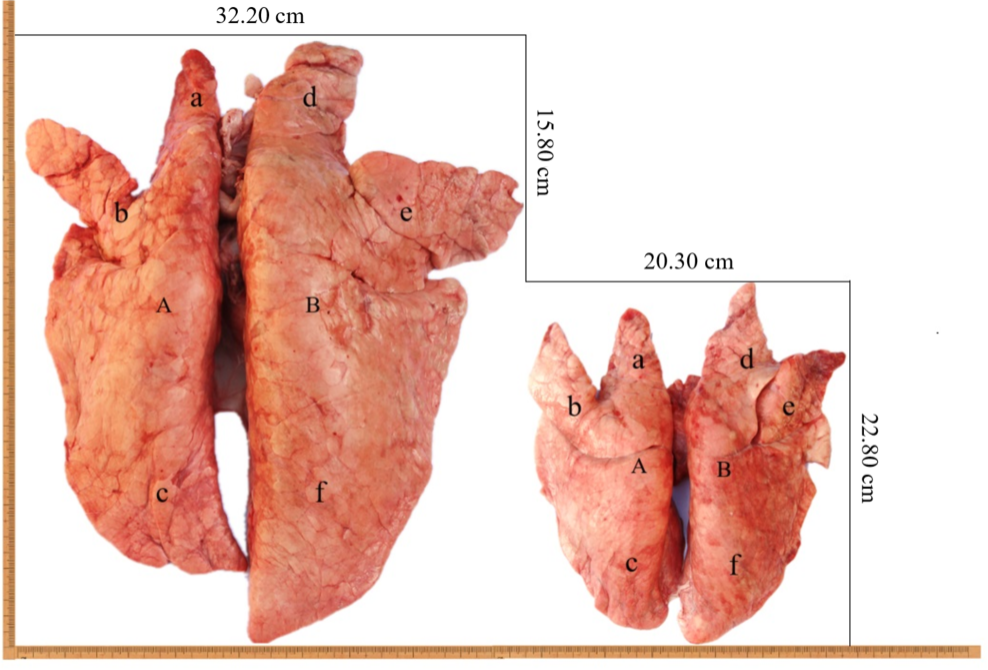
The lungs of Tibetan pigs (right) and Landrace pigs (left). A: left lung; B: right lung; a: left anterior lobe; b: left middle lobe; c: left rear lobe; d: right anterior lobe; e: right middle lobe; f: right rear lobe.

**Table 1:** Pulmonary morphological indicators.

Ratio	Breeds			
	TGN	TJC	LYD	LJC
Lung weight / body weight	0.0085 ${}^{a}$ ± 0.0010	0.0084 ${}^{a}$ ± 0.0010	0.0063 ${}^{b}$ ± 0.0010	0.0067 ${}^{b}$ ± 0.0001
Density	0.0008 ${}^{a}$ ± 0.0001	0.0008 ${}^{a}$ ± 0.0001	0.0005 ${}^{b}$ ± 0.0001	0.0005 ${}^{b}$ ± 0.0001
Major axis / minor axis of the lung	1.8936 ${}^{b}$ ± 0.0587	1.8026 ${}^{b}$ ± 0.1024	2.1216 ${}^{a}$ ± 0.1184	2.0077 ${}^{\mathrm{ab}}$ ± 0.1255

Note: because the Tibetan and Landrace pigs exhibited major differences in body weight, we compared the ratio of the lung weight to the body weight. Values with the same superscripts in the same row did not differ significantly ( P>0.05 ), and values with different superscripts in the same row differed significantly ( P<0.05 ).

**Figure 2:**
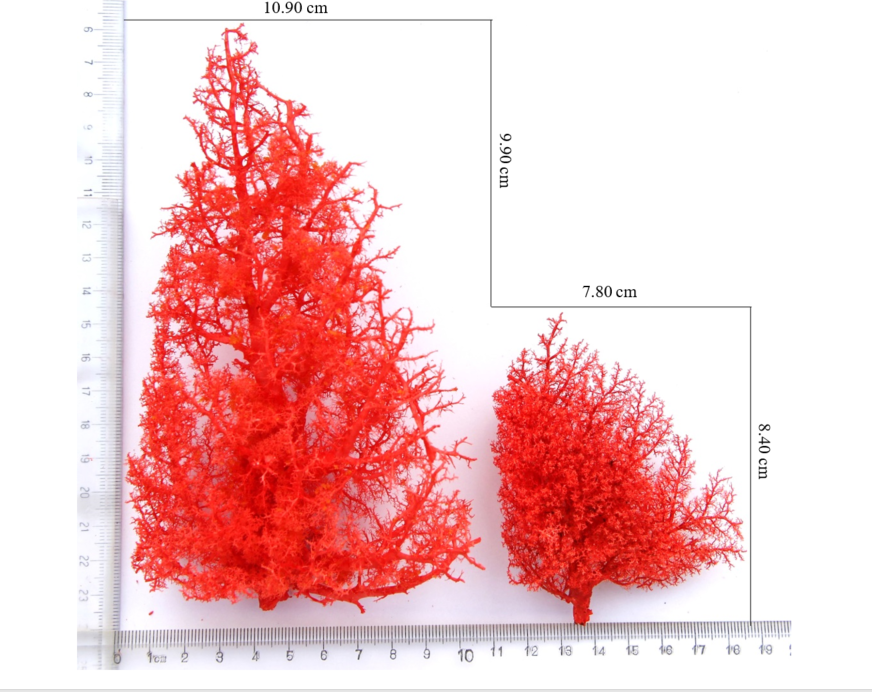
Morphological features of the pulmonary artery. Tibetan pig cast specimens of the pulmonary artery are on the left, and Landrace pig cast specimens of the pulmonary artery are on the right.

**Table 2:** Diameters of the left pulmonary artery of pigs.

Part	Branches	Average diameter or weight			
		TGN	LYD	TJC	LJC
		(3000 m)	(2500 m)	(1000 m)	(1000 m)
Ratio (major / minor axis)		1.3500	1.6900	1.4400	1.7500
Anterior lobe	Trunk	0.1084 ${}^{a}$ ± 0.0210	0.0513 ${}^{b}$ ± 0.0029	0.0914 ${}^{a}$ ± 0.0165	0.0444 ${}^{b}$ ± 0.0021
	First-level branch	0.0215 ${}^{a}$ ± 0.0040	0.0098 ${}^{b}$ ± 0.0005	0.0175 ${}^{a}$ ± 0.0026	0.0085 ${}^{b}$ ± 0.0003
	Second-level branch	0.0102 ${}^{a}$ ± 0.0020	0.0048 ${}^{b}$ ± 0.0003	0.0086 ${}^{a}$ ± 0.0016	0.0042 ${}^{b}$ ± 0.0002
	Third-level branch	0.0088 ${}^{a}$ ± 0.0003	0.0025 ${}^{b}$ ± 0.0001	0.0085 ${}^{a}$ ± 0.0004	0.0023 ${}^{b}$ ± 0.0001
	Fourth-level branch	0.0058 ${}^{a}$ ± 0.0004	0.0014 ${}^{b}$ ± 0.0002	0.0061 ${}^{a}$ ± 0.0008	0.0014 ${}^{b}$ ± 0.0001
Middle lobe	Trunk	0.0621 ${}^{a}$ ± 0.0121	0.0294 ${}^{b}$ ± 0.0016	0.0508 ${}^{a}$ ± 0.0103	0.0254 ${}^{b}$ ± 0.0012
	First-level branch	0.0092 ${}^{a}$ ± 0.0019	0.0043 ${}^{b}$ ± 0.0003	0.0077 ${}^{a}$ ± 0.0014	0.0036 ${}^{b}$ ± 0.0003
	Second-level branch	0.0081 ${}^{a}$ ± 0.0007	0.0021 ${}^{b}$ ± 0.0001	0.0082 ${}^{a}$ ± 0.0006	0.0022 ${}^{b}$ ± 0.0003
	Third-level branch	0.0035 ${}^{a}$ ± 0.0007	0.0017 ${}^{b}$ ± 0.0001	0.0030 ${}^{a}$ ± 0.0005	0.0014 ${}^{b}$ ± 0.0001
	Fourth-level branch	0.0027 ${}^{a}$ ± 0.0006	0.0010 ${}^{b}$ ± 0.0000	0.0028 ${}^{a}$ ± 0.0003	0.0007 ${}^{b}$ ± 0.0000
Rear lobe	Trunk	0.0248 ${}^{a}$ ± 0.0048	0.0120 ${}^{b}$ ± 0.0009	0.0209 ${}^{a}$ ± 0.0038	0.0107 ${}^{b}$ ± 0.0018
	First-level branch	0.0198 ${}^{a}$ ± 0.0038	0.0092 ${}^{b}$ ± 0.0005	0.0165 ${}^{a}$ ± 0.0021	0.0035 ${}^{b}$ ± 0.0001
	Second-level branch	0.0098 ${}^{a}$ ± 0.0014	0.0041 ${}^{b}$ ± 0.0001	0.0087 ${}^{a}$ ± 0.0010	0.0065 ${}^{b}$ ± 0.0001
	Third-level branch	0.0070 ${}^{a}$ ± 0.0003	0.0022 ${}^{b}$ ± 0.0000	0.0067 ${}^{a}$ ± 0.0003	0.0020 ${}^{b}$ ± 0.0000
	Fourth-level branch	0.0042 ${}^{a}$ ± 0.0004	0.0012 ${}^{b}$ ± 0.0001	0.0047 ${}^{a}$ ± 0.0004	0.0011 ${}^{b}$ ± 0.0001

Notes: values with the same superscripts in the same row did not significantly differ ( P>0.05 ), and values with different superscripts in the same row differed significantly ( P<0.05 ).

**Table 3:** Blood physiological indexes of the pigs.

Index	Unit	Breeds			
		TGN	LYD	TJC	LJC
		(3000 m)	(2500 m)	(1000 m)	(1000 m)
RBC	× 10 ${}^{12}$ /L	7.25 ${}^{\mathrm{ab}}$ ± 0.74	7.94 ${}^{a}$ ± 0.27	6.34 ${}^{b}$ ± 0.81	7.23 ${}^{\mathrm{ab}}$ ± 0.55
HGB	g/L	148.33 ${}^{a}$ ± 3.46	152.00 ${}^{a}$ ± 5.03	112.67 ${}^{c}$ ± 5.51	128.00 ${}^{b}$ ± 5.00
HCT	%	53.37 ${}^{a}$ ± 3.73	48.13 ${}^{b}$ ± 1.22	37.27 ${}^{c}$ ± 1.57	45.80 ${}^{b}$ ± 2.08
MCV	fL	73.77 ${}^{a}$ ± 4.21	60.63 ${}^{b}$ ± 1.76	59.20 ${}^{b}$ ± 5.47	63.47 ${}^{b}$ ± 3.43
MCH	pg	20.57 ${}^{a}$ ± 1.14	18.70 ${}^{\mathrm{ab}}$ ± 0.66	17.93 ${}^{b}$ ± 1.64	18.63 ${}^{\mathrm{ab}}$ ± 0.61
MCHC	g/L	302.67 ${}^{\mathrm{ab}}$ ± 4.04	308.33 ${}^{a}$ ± 2.52	279.00 ${}^{c}$ ± 5.29	294.00 ${}^{b}$ ± 6.08
RDW-CV	%	18.03 ${}^{a}$ ± 0.70	16.60 ${}^{\mathrm{ab}}$ ± 0.8	16.20 ${}^{b}$ ± 0.44	16.70 ${}^{\mathrm{ab}}$ ± 1.25
RDW-SD	fL	55.60 ${}^{a}$ ± 3.10	41.93 ${}^{b}$ ± 1.89	40.07 ${}^{b}$ ± 2.73	44.27 ${}^{b}$ ± 3.33
Hb ${}^{*}$	g/L	14.73 ${}^{a}$ ± 0.23	14.67 ${}^{a}$ ± 0.40	12.10 ${}^{c}$ ± 0.44	13.27 ${}^{b}$ ± 0.58

Notes: values with the same superscripts in the same row did not differ significantly ( P>0.05 ), and values with different superscripts in the same row differed significantly ( P<0.05 ).

**Figure 3:**
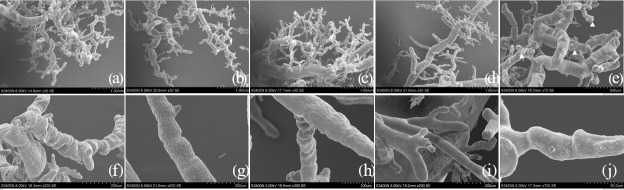
Distribution and superficial features of the arterioles in the left lung. **(a)** The arteriole distribution of the LYD group ( × 30). **(b)** The arteriole distribution of the TGN group ( × 20). **(c)** The arteriole distribution of the TJC group ( × 30). **(d)** The arteriole distribution of the LJC group ( × 30). **(e)** Microvascular terminal, with a triangle mark ( × 70). **(f)** Imprints of the endothelial nuclei of the LYD group ( × 200). **(g)** Imprints of the endothelial nuclei of the TGN group ( × 200). **(h)** Imprints of the endothelial nuclei of the TJC group ( × 300). **(i)** Imprints of the endothelial nuclei of the LJC group ( × 200). **(j)** Precapillary arterioles ( × 700).

**Figure 4:**
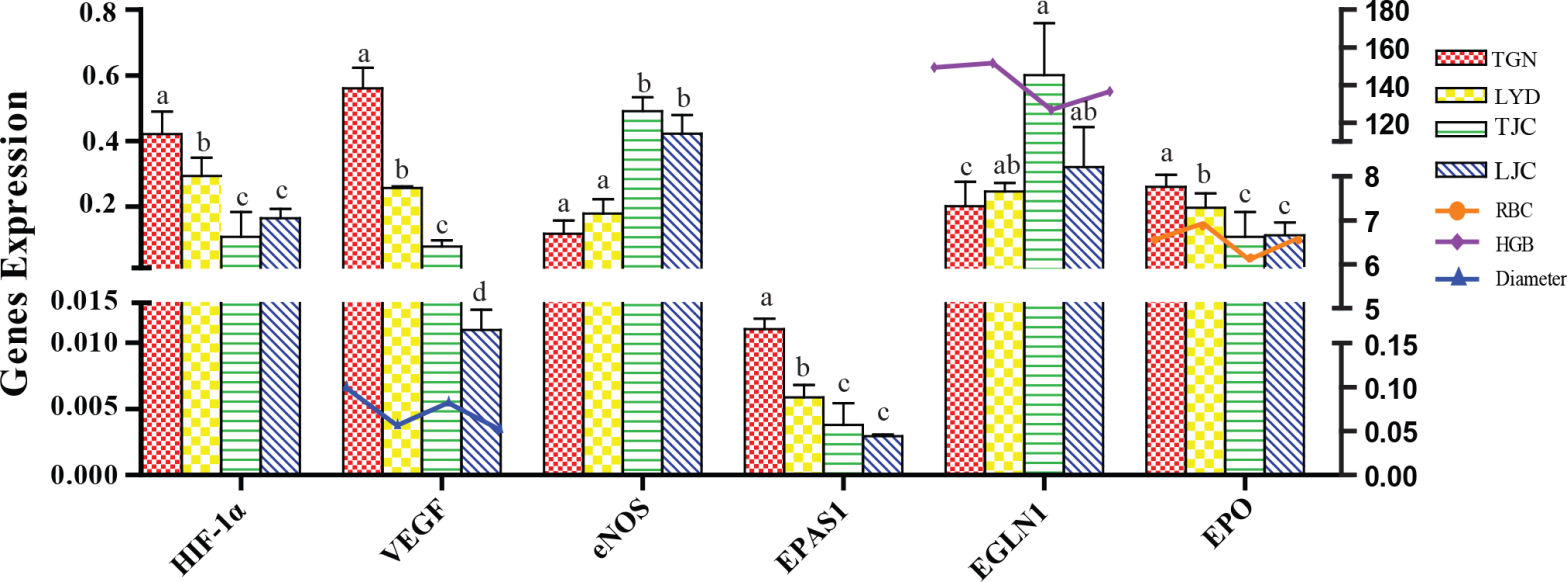
Expression levels of *HIF*- *1* α , *EPAS1*, *EPO*, *VEGF*, *eNOS* and *EGLN1* in the lungs of the four types of pigs and the relationship between *VEGF*, *EPO* and *EGLN1* and diameter, RBCs and HGB. Note: the different letters above the bars indicate that the expression levels differed significantly ( P<0.05 , mean ± SE), and the same letters indicate that the levels did not differ significantly ( P>0.05 , mean ± SE) in different breeds.

## Data Availability

The data are available from the corresponding author upon request.

## 1 Introduction

Hypoxia can trigger a series of stress reactions, such as metabolic disorders and tissue and cytomembrane damage (Jeong et al., 2018; Gan et al., 2019; Yanyu et al., 2019). To adapt to high-altitude environments, indigenous animals have developed unique physiological mechanisms during the long process of evolution and have acquired stable structural and functional characteristics to ensure a sufficient oxygen supply in their tissues and cells under hypoxic conditions (Ishikawa et al., 2019; Patra et al., 2019; Zhao et al., 2019). Tibetans have larger lungs and lower hemoglobin (HGB) concentrations than other groups, and the lungs of yaks and Tibetan sheep have larger pulmonary alveolar areas per unit area, thicker diameters, more branches and denser pulmonary artery networks than those of low-altitude animals (Wang et al., 2020; Moore et al., 2001; Qi et al., 2019). Physiological studies have shown that Tibetan pigs, as plateau mammals native to the Tibetan Plateau, have evolved physiological adaptations, such as increases in the size and strength of the heart and lung in response to high-altitude hypoxic conditions (Ai et al., 2014). Tibetan pigs have a thicker alveolar diaphragm and more capillaries than Landrace pigs (Bai et al., 2012). The red blood cell (RBC) number and HGB level of Tibetan pigs at high altitudes are significantly higher than those of low-altitude pigs (Kong et al., 2014).

Hypoxic adaptability is also reflected by the level of hypoxia-related gene regulation (Ishikawa et al., 2019; Yu et al., 2018; Bi et al., 2017; Cheng et al., 2016). The genes involved in the hypoxia-induced pathway include the positive regulatory factors, hypoxia-inducible factor (*HIF*- *1* α ), vascular endothelial growth factor (*VEGF*), endothelial PAS domain-containing protein 1 (*EPAS1*), endothelial nitric oxide synthase (* eNOS*), erythropoietin (*EPO*) and the negative regulatory factor egl nine homolog 1 (*EGLN1*), and their regulated expression can lead to large increases in hypoxia-related substances (Ishikawa et al., 2019; Mohamed et al., 2019; Ma et al., 2019). At present, the mechanisms related to the adaptation of Tibetan pigs to high altitude have not been fully elucidated. The architecture of the pulmonary arteries of Tibetan pigs has rarely been studied. In this study, we collected pulmonary tissue samples from Tibetan pigs living at 3000 and 1000 m and Landrace pigs living at 2500 and 1000 m. The morphology of pulmonary artery cast specimens, the values of blood physiological indexes and the expression of hypoxia-responsive genes were compared to help elucidate the biological mechanism underlying the adaptation of Tibetan pigs to high-altitude hypoxic conditions.

## 2 Materials and methods

### 2.2 Sample collection

Forty-four fresh lungs from two breeds – Tibetan pigs that live in Gannan (TGN, altitude of 3000 m) and Jingchuan (TJC, altitude of 1000 m, moved from Gannan) and Landrace pigs that live in Yongdeng (LYD, altitude of 2500 m) and Jingchuan (LJC, altitude of 1000 m) in Gansu – were collected. We bred 12 male piglets from the population with a similar weight and non-genetic relationship for every group. We randomly selected three pigs from each group to collect the left lower lobes of the lung from adult male pigs at the age of 6 months within 1 h after the pigs were slaughtered, and the samples were stored immediately in liquid nitrogen for later RNA extraction; six lungs were cleaned to create three-dimensional architectures. Samples of venous blood were collected.

### 2.3 Generation of casts and measurement of the diameters

The arteries of the left lung were perfused with ABS (acrylonitrile butadiene styrene) agent using the following protocol: first, the blood of the lung was repeatedly squeezed from the margin to the aorta to expel congestion. Second, a needle was inserted and fixed from the left pulmonary artery, and the ABS agent (12 %) was injected. The sample was placed in cold water for 1 h and reperfused several times. The sample was then solidified and soaked in 31 % concentrated hydrochloric acid for 2 weeks to obtain a cast of the whole specimen. Photographs of the morphological specimens were obtained with a digital camera, and their anatomic characteristics were observed and compared among different breeds.

The diameters of the arteries and arterioles in the same parts of the lungs of Tibetan and Landrace pigs at different altitudes were measured and recorded using Vernier calipers, and the results were calculated as the average from three parts, namely, the root, middle and terminal sections. Microarterioles sampled from the same part of the lung specimen were selected and images were taken. After the samples were cleaned and dried by ultrasonication, gold plating was added using an ion coating machine (JMB – 3500 va). The samples were observed using a scanning electron microscope (Hitachi S – 3400 n) to select different regions, and the microvascular diameters were measured using Image J software.

### 2.4 Measurement of blood physiological indexes

A hematology analyzer (GRT-6008) was used to measure the blood parameters of 12 pigs.

### 2.5 Quantitative real-time PCR (qRT-PCR) analysis of the expression of hypoxia-related genes

Total RNA was extracted using the total RNA extraction kit from Biotech (TianGen, China), and the HiScript^®^ II 1st strand cDNA synthesis kit (Vazyme, China) was used for the reverse transcription of cDNA. Subsequently, ChamQ™ Universal SYBR^®^ qPCR Master Mix (Vazyme) was used for real-time fluorescence quantitative analysis. Primer sequences for the *HIF*- *1* α , *EPAS1*, *EPO*, *VEGF*, *eNOS*, *EGLN1* and *GAPDH* (reference) genes were used for qRT-PCR (Table S1). The transcript abundance was calculated using the following formula: 2( $\Delta^{\mathrm{Ct}}$ Test – $\Delta^{\mathrm{Ct}}$ Control). The data were analyzed using the 2 ${}^{-\mathrm{Ct}}$ method.

### 2.6 Statistical analysis

The differences in the pulmonary artery morphology of the lungs in Tibetan and Landrace pigs were analyzed and compared. The SPSS 19.0 software was used for the data analyses. Intergroup comparisons were performed by one-way ANOVA, and Duncan's method was used for multiple comparisons. The diameters, blood physiological indexes and gene expression levels in the pulmonary arteries of Tibetan pigs and Landrace pigs were analyzed, and P<0.05 indicated a significant difference.

## 3 Results

### 3.1 Differences in lung morphologies

In this study, the analysis of the pulmonary morphology revealed some differences in size and type between Tibetan (TGN and TJC) and Landrace pigs (LYD and LJC) (Fig. 1, Table 1). Although the lungs of Tibetan pigs were markedly smaller than those of Landrace pigs, the weight of adult Tibetan pigs was also lower than that of Landrace pigs; thus, we used the ratio of lung tissue to individual weight to represent the relative weight of the lungs. Surprisingly, the ratio obtained for Tibetan pigs was significantly higher than that found for Landrace pigs ( P<0.05 ). The density of Tibetan pigs was significantly higher than that of Landrace pigs ( P<0.05 ); in contrast, the ratio of the major axis to the minor axis found for Tibetan pigs was lower than that obtained for Landrace pigs. These findings indicated that the lungs of Tibetan pigs were heavier and wider than those of Landrace pigs.

### 3.2 Differences in the anatomical architecture and diameter of the pulmonary artery

The anatomical structure of the pulmonary artery was clearly observed using the cast specimens (Fig. 2). The number, relative position and direction of the aorta and its branches were clearly discernible. An analysis of the pulmonary artery specimens from Tibetan and Landrace pigs indicated that the distribution pattern of the arteries was shaped like a tree branch. The pulmonary artery is divided into three parts, including the ascending branch and the downward branch, which are shaped like an arch. Several flexible arterioles are divided at different angles; the first branch exhibits the smallest angle, and the arterioles, which are shaped like a tree branch, show the largest angle. The comparison of the left lungs of Tibetan and Landrace pigs revealed that the numbers of first pulmonary arteries in these pigs were 23–27 and 28–32, respectively. In addition, Tibetan pigs had more and denser arterioles than Landrace pigs, and the pulmonary artery branch of Tibetan pigs presented a larger angle at the same site than that of Landrace pigs.

We compared the relative diameters of the pulmonary arteries of the four groups and branches based on the weight of Tibetan and Landrace pigs at different altitudes. The results showed similar arterial distributions and variations in diameter between the groups, and the diameter decreased with increases in the branch level. The major / minor axis ratio of Tibetan pigs was lower than that of Landrace pigs. The average diameter of the main pulmonary artery and branches of Tibetan pigs was significantly larger than that of Landrace pigs ( P<0.05 ), and the diameters of the high-altitude pigs were larger than those at low altitudes ( P>0.05 ).

### 3.3 Characteristics of arterioles observed by scanning electron microscopy

A scanning electron microscopy analysis revealed that the pulmonary artery surface of Tibetan and Landrace pigs was rough and had a “dendritic” shape (Fig. 3a–d). A three-dimensional analysis revealed that each trunk produced second- to fourth-grade branches at different angles. Each branch had an increasing number of branches from the aorta to the arterioles. In the arterial process, the branch twisted and turned at a certain angle, and a smaller diameter was associated with increased folding. The arterioles with diameters of less than 0.42 mm had branches at a 90 ${}^{\circ }$ angle with obtuse branches at the end (Fig. 3e). The left pulmonary arterioles with a 72.64–123.89 µ m diameter on the surface were distributed in endothelial cell indentations, which were distributed unevenly and largely in LYD, evenly and deeply in TGN, and poorly and shallowly in TJC and were not found in LJC. Most endothelial cell indentations were shaped like a boat or footprint, and the length and width were approximately 6.55–9.88 and 3.22–4.51 µ m, respectively (Fig. 3f–i). However, the surface dents of the precapillary arterioles with diameters of approximately 18.23–22.23 µ m were smooth in all four sets of pigs. We observed a deeper annular constriction on the surface of micro-vessels in the high-altitude pigs than in the low-altitude pigs.

### 3.4 Determination of blood biochemical indexes

The measurement of various blood biochemical indicators related to the oxygen concentration in the pigs showed that the RBC number of LYD was significantly larger than that of TJC. The HGB, corpuscular hemoglobin concentration (MCHC) and Hb ${}^{*}$ values of the high-altitude pigs were significantly higher than those of the low-altitude pigs. The hematocrit (HCT), mean corpuscular volume (MCV), mean corpuscular hemoglobin (MCH), red cell volume distribution width (RDW-CV) and red blood cell distribution width standard deviation (RDW-SD) values of the TGN group were significantly larger than those of the other groups.

### 3.5 Expression of genes correlated with hypoxia

The qRT-PCR analysis confirmed that the expression levels of *HIF*- *1* α , *EPAS1*, *EPO*, *VEGF*, *eNOS* and *EGLN1* differed between the different breeds (Fig. 4). The expression levels of *HIF*- *1* α , *EPAS1*, *EPO* and*VEGF* in the lungs of TGN and LYD were significantly higher than those in the lungs of TJC and LJC ( P<0.05 ), and the highest levels of these genes were found in the TGN group. The expression of *VEGF* was highest in the TGN group and lowest in the LJC group, and the diameter of the pulmonary artery exhibited a similar trend. A comprehensive analysis showed that the expression levels of *EPO* and RBC were higher in the high-altitude pigs than in the low-altitude pigs. Notably, the expression of *eNOS* in the TJC and LJC groups was significantly higher than that in the TGN and LYD groups, and the expression of *EGLN1* in TJC was significantly higher than that in TGN ( P<0.05 ) and negatively correlated with the HGB.

## 4 Discussion

### 4.1 Structure and function of the pulmonary artery of pigs

To adapt to the hypoxic environment of the plateau, the lungs of plateau animals gradually increased in size during evolution, which is beneficial for enhancing the blood capillary and respiratory area. These findings were also found in our studies (Bai et al., 2012). The presence of multiple branches and a large respiratory region increase the area of air exchange in the lungs, which is necessary for increased lung ventilation and a higher gas exchange rate in animals living in a high-altitude hypoxic environment. The diameter of the pulmonary arteries that supply blood to the lungs of Tibetan sheep is larger than that of Small-tailed Han sheep (Jia et al., 2018). In addition, the contents of capillaries in the alveolar septum of plateau animals, including yak and Tibetan sheep, were richer than those of plain animals. Developed blood vessels can supply sufficient blood to the lungs to increase the number of alveoli and provide rich nutrients for lung growth and development, which is conducive to accelerating the transport of blood oxygen (Qi et al., 2019; Jia et al., 2018; Zhang et al., 2015a). The developed lungs and arteries of Tibetan pigs can reduce the vascular resistance and increase the blood flow, which is conducive to improving the flow of blood and oxygen to the pulmonary artery system to alleviate the insufficient supply of oxygen at high altitudes.

Vascular endothelial cells are widely distributed in various tissues and are the only cells in contact with blood (Yang et al., 2019). Vascular smooth muscle cells, which are generally shaped like “tapers”, play an important role in regulating the blood flow rate and maintaining vascular tension (Wang et al., 2015). Some studies have found that the surfaces of the cerebral and heart arteries of yak and Tibetan sheep resemble “bark”, and the indentation on the surface is shaped like a taper (He et al., 2007). Vascular endothelial cells with a lamellar and lamelliform appearance are conducive to the absorption of liquid from plasma and its transporting to tissues. Large finger-like features are used to buffer rapid blood flow in large vessels and facilitate material exchange (Sturtzel et al., 2017; Li et al., 2014). Due to their exposure to low temperature, plateau animals often appear sluggish and show constricted peripheral blood vessels. We found that the lungs of Tibetan pigs present a greater arteriolar distribution, larger diameter, more capillaries and more dents on the surface than those of Landrace pigs. These findings indicated that Tibetan pigs exhibit an increased blood supply in the lungs, which is conducive to overcoming the obstruction of the respiratory system caused by the high-altitude and low-oxygen environment.

### 4.2 Blood biochemical indexes of pigs

This study showed that the RBC and HGB concentrations of pigs at high altitudes (TGN and LYD) were significantly higher than those at low altitudes (TJC and LJC). The increase in RBCs induced by a higher altitude is conducive to overcoming the high-altitude and low-oxygen environment, which is consistent with the results from an immigration test of Tibetan pigs and is conducive to improving the body's tolerance to hypoxic environments (Zhang et al., 2015b).

Compared with lowland cattle, domestic yaks have developed physiological features adapted to high-altitude hypoxic conditions, such as a thinner alveolar septum and a thinner blood–air barrier (Zhou et al., 2015). The native highlander Tibetans also acquired similar physiological features, such as an enhanced hypoxic and hypercapnic ventilatory responsiveness, an improved lung diffusion capacity, minimal hypoxic pulmonary hypertension, high levels of exhaled nitric oxide (NO), and low levels of HGB (Qi et al., 2015) . In addition, both domestic yaks and Tibetans maintain a normal blood HGB level compared with that of lowland cattle and Han populations, respectively. These findings suggest that highland Tibetans and domestic yak likely have similar strategies (red cell overproduction) to protect themselves from high-altitude polycythemia (Zhang et al., 2015; Beall et al., 1998). Earlier studies have proposed that high HGB concentrations are conducive to long-term living in a hypoxic environment (Beall et al., 2002). These physiological features might facilitate efficient blood flow for oxygen delivery under hypobaric hypoxia. Therefore, HGB is considered the key factor for individual fitness in hypoxic environments. The blood indexes of Tibetan pigs living in plateau (3000 m) and flatland habitats (1000 m) were measured, and these measurements revealed that those at high altitude presented significant reductions in the hematocrit level, average erythrocyte volume and erythrocyte distribution width and decreases in the leucocyte and average erythrocyte HGB levels compared with those at low altitude (Xu et al., 2019). HGB, a complex allosteric iron-containing protein in vertebrate RBCs, is formed from the combination of heme and globin, which is a key indicator of respiratory movement and could transport oxygen and carbon dioxide and maintain the acid–base balance in blood (Wajcman et al., 2002).

High HGB concentrations might be an adaptive mechanism linked to hemodynamic mechanisms that induce blood thickening, affect the transport of oxygen in blood and eventually cause chronic altitude sickness (CMS) (Beall et al., 1998, 2002). Therefore, HGB was once considered the key factor of fitness in a hypoxic environment. The RBC, HGB and hematocrit levels might be responsible for the compensatory increase in RBCs in hypoxic environments through the proliferation and synthesis of HGB, which improves the oxygen-carrying ability of pigs and allows their migration to a plateau environment from plains through their adaption to high-altitude hypoxic environments (Xu et al., 2019; Wajcman et al., 2002; Zhang et al., 2019). A higher level of HGB is associated with a higher oxygen affinity and a higher tolerance to hypoxia. A series of indicators, such as the measured reductions in the hematocrit level and erythrocyte volume, maintained a low blood viscosity, which is conducive to the smooth circulation of the blood and a reduction in the heart burden (Yang et al., 2017; Tang et al., 2019). All of these factors could provide an important hematological basis for the mechanism underlying the adaptation of Tibetan pigs to high altitude.

### 4.3 Differential expression of hypoxia-related genes in the lung and their associations with the morphology and physiology of pigs

Tibetan pigs can survive on the plateau environment due to their adaptation to hypoxic conditions, and the normal functioning of the lung is mainly regulated by angiogenesis, cellular energy metabolism and the expression of hypoxia-related genes. These processes are generally associated with HIF, which is a heterodimer consisting of the *HIF*- *1* α and *HIF*- *1* β subunits and a member of the basic helix–loop–helix PER–ARNT–SIM (bHLH-PAS) protein family (Chertok et al., 2018).

The *HIF*- *1* α subunit degrades under normoxic conditions but is stable under hypoxic conditions, whereas the *HIF*- *1* β subunit is constitutively expressed. *HIF*- *1* α and *EPAS1* can regulate hundreds of genes, such as the positive regulators *VEGF*, *EPO* and *eNOS* and the negative regulator *EGLN1* (Yu et al., 2018; Bhattarai et al., 2018; Yu et al., 2020). The *EGLN1* gene is an important oxygen-sensing gene [4], and a decreased hydroxylation activity of *EGLN1* could lead to gradual increases in the expression of *HIF*- *1* α and *HIF*- *2* α . *EPAS1*, which is the only gene located within a 70 kb region on chromosome 3 in Tibetan pigs, encodes the *HIF*- *2* α protein and is responsible for an increased regulation of many hypoxia-induced genes, including *EPO*, *VEGF* and *eNOS* (Yu et al., 2018; Xu et al., 2018). Hypoxia is the main cause of *EPO* secretion. As a traditional indicator of erythropoiesis and HGB, *EPO* is the first hypoxic fitness indicator proposed, and the pig *EPO* gene is located on chromosome 3 and consists of four introns and five exons. Elevated EPO amplifies the proliferation and differentiation of RBC precursors and increases HGB levels (Deji et al., 2015; Auzmendi et al., 2020). *VEGF*, as a target gene of *HIF*- *1* α , can promote endothelial cell proliferation and neo-vascularization and increase vascular permeability (Yu et al., 2018). *HIF*- *1* α is positively correlated with *VEGF* and increases with increases in hypoxia at high altitudes (Dzhalilova et al., 2018). In our studies, the high expression of *HIF*- *1* α , *EPAS1*, *EPO* and the normal expression of *VEGF* and *eNOS* in the high-altitude pigs were consistent with the large artery diameter and the high number of vascular branches found in these pigs. Increases in the hematocrit level and the blood viscosity can improve the oxygen-carrying capacity of blood as well as the symptoms of hypoxia. NO, which is expressed in multiple cell types as a vasodilator, and *eNOS* produced by NO can regulate the vasoconstriction and dilatation of blood vessels, which could induce the relaxation of smooth muscles and the dilation of arteries to regulate the blood pressure and blood flow distribution (Ghosh et al., 2018). *EPAS1* can enhance the expression of *eNOS* and eventually lead to an increase in the NO levels, and reduced oxygen levels and increased blood flow facilitate the supply of oxygen to the lungs (Yang et al., 2019). Some studies have shown that long-term exposure to a hypoxic environment inhibits *eNOS* activity, reduces *eNOS* expression in mouse lung tissues, decreases the production and release of NO, weakens the vasodilatory effects, promotes the proliferation of pulmonary vascular endothelial cells and smooth muscle cells, enhances pulmonary vascular remodeling, and increases the pulmonary artery pressure (Janaszak-Jasiecka et al., 2018). The activity and expression of *eNOS* were inhibited in the lung tissues of Landrace pigs after migration from the lowland to the plateau environment, and the difference in *eNOS* expression between Tibetan and Landrace pigs was obvious and consistent with the results of our study (Zhang et al., 2013). Recent studies have also indicated that *EGLN1* exhibits a significant negative correlation with the HGB levels. The high expression of *EGLN1* in lung tissues of Tibetan and Landrace pigs at high altitudes is negatively correlated with the HGB concentration (Zhang et al., 2013). Therefore, the expression of genes related to oxygen adaptation, vasodilation and an increase in the number of capillaries, RBC count and HGB concentration might be the biological regulatory mechanism underlying the adaptation of Tibetan pigs to the high-altitude and low-oxygen environment of the plateau.

## 5 Conclusions

This study demonstrates that the lungs of Tibetan pigs are heavier and wider than those of Landrace pigs and that Tibetan pigs have a higher number of and denser arteries and arterioles than Landrace pigs. The analysis of the lung structure revealed that the high-altitude Tibetan pigs had more developed lungs and abundant arteries to ensure their respiration in a low-oxygen environment. In addition, the HIF-1 signaling pathway might be one of the major factors that influence the RBC count, HGB level and number of arterioles, which might be regulated through the environment. The findings also show that the regulation of the lung function of Tibetan pigs in a hypoxic environment is manifested at various levels to ensure breathing under extreme environmental conditions.
