# Supplementary material for: Characteristics of Tibetan pig lung tissue in response to a hypoxic environment on the Qinghai–Tibet Plateau
Source: Arch Anim Breed. 2021 Jun 28;64(1):283–92. doi: 10.5194/aab-64-283-2021 (PMC8253108; doi:10.5194/aab-64-283-2021)
Supplement: The supplement related to this article is available online at: https://doi.org/10.5194/aab-64-283-2021-supplement. [file aab-64-283-supplement.pdf]

Supplement of Arch. Anim. Breed., 64, 283–292, 2021
https://doi.org/10.5194/aab-64-283-2021-supplement
© Author(s) 2021. CC BY 4.0 License.

Open Access
Archives Animal Breeding

Supplement of

Characteristics of Tibetan pig lung tissue in response to a hypoxic environment on the Qinghai–Tibet Plateau

Yanan Yang et al.

Correspondence to: Shengguo Zhao (zhaosg@gsau.edu.cn)

The copyright of individual parts of the supplement might differ from the article licence.

Supplementary

S1 Table

Information on primers

Genes	Primer sequences (5'-3')	Tm/°C
HIF-1*α*	F: 5'-TTGAAGATGAAATGAAGGCACAGA-3'<br>R: 5'-ATGGTCGCACGGATGAGTAAA-3'	60
*EPAS*1	F: 5'-TGGACAAGGCCTCCATCAT-3'<br>R: 5'- CAATGAAACCCTCCAAGGC-3'	60
EPO	F: 5'-TCCTGGAGAGGTACATCTT-3'<br>R: 5'-AGGCATAGAAGTTAACCTT-3'	60
VEGF	F: 5'-GCCGTCCAATCGAGACCCT-3'<br>R: 5'-CATGGCGATGTTGAACTCCTC-3'	60
eNOS	F: 5'-CCCAGAGAATGGAGAGAGTT-3'<br>R: 5'-TATTGAAGCGGATTTTGTAA-3'	60
EGLN1	F: 5'-CGATAAGATCACCTGGATCGA-3'<br>R: 5'-TTGTAGTTGCCCAGTTTGCCG-3'	60
GAPDH	F: 5'-GGTCACCAGGGCTGCTTTTA-3'<br>R: 5'-CCTTGACTGTGCCGTGGAAT-3'	60
